# What is a season to an oryx? Movement rates identify three seasons for scimitar-horned oryx reintroduced into their native range

**DOI:** 10.1186/s40462-025-00536-7

**Published:** 2025-07-29

**Authors:** Kristen Whyle, Katherine Mertes, Ricardo Pusey, Saeed Al Romaithi, Mohammed Al Remeithi, Ahmed Esmaeil Alsayed Alhashmi, Mahamat Hassan Hatcha, Ali Ngare Walsoumon, Abdramane Hamid Chaibo, Taboye Abdelkerim, Habib Ali, Oumar Mahamat Annadif, Kher Issaka, Mahamat Ali, Marc Dethier, John Newby, Melissa Songer

**Affiliations:** 1 Smithsonian’s National Zoo and Conservation Biology Institute, Front Royal, VA USA; 2 Terrestrial & Marine Biodiversity Sector, Environment Agency–Abu Dhabi, Abu Dhabi, UAE; 3 Ministère de l’Environnement de l’Eau, de la Pêche et du Développement Durable, N’Djamena, Chad; 4 Sahara Conservation, Saint-Maur-Des-Fossés, France; 5 Department of Fish and Wildlife Sciences, University of Idaho College of Natural Resources, Moscow, ID USA

**Keywords:** Animal movement, Movement behavior, Seasonality, Conservation translocation, Reintroduction, Generalized additive mixed model, Reproductive state

## Abstract

**Background:**

Abundant evidence exists that mobile animals exhibit different movement behavior during different seasons, especially in landscapes with strong seasonal variation in climate and resource availability. Quantifying seasonal movement dynamics is critical for making accurate inferences and appropriate recommendations for species conservation and landscape management. Using empirical approaches to characterize seasonal variation in animal movement minimizes assumptions about the timing of seasonal transitions, environmental proxies, and effects of spatiotemporal variation.

**Methods:**

We calculated 57,255 mean daytime hourly movement rates for 104 scimitar-horned oryx (*Oryx dammah*) released into a large protected area in central Chad from 2016 to 2022. We used these movement data to build generalized additive mixed models of movement rates over a generic calendar year to detect potential seasonal variation in oryx movement behavior.

**Results:**

Our final model indicated that reintroduced oryx experience three seasons per year, exhibiting dramatically lower daytime movement rates during the hot, dry season and higher movement rates during the rainy and cool, dry seasons. Reproductive status also affected oryx movement rates, notably females 1–4 months into pregnancy.

**Conclusions:**

Captive-born oryx exhibited transitions in movement behavior aligned with regionally characteristic seasonal variation, a promising indicator for an ongoing reintroduction effort. Females 1–4 months pregnant, particularly those accompanied by neonates, exhibited consistently elevated daytime movement rates, suggesting substantial energy allocation to foraging in early pregnancy. The three seasons delineated by this study will be used to manage the reintroduced oryx population, for example to identify priority areas and time periods for enhanced monitoring and enforcement actions, as well as to investigate the potential re-emergence of historical seasonal migrations.

**Supplementary Information:**

The online version contains supplementary material available at 10.1186/s40462-025-00536-7.

## Background

Many ecosystems exhibit strong seasonal variation in climate and resource availability, and species living in these systems often alter their behavior to maximize fitness in each season. For example, many temperate and arctic ungulates reduce overall movement and activity during winter months, likely to conserve energy and minimize loss of condition, then increase movement rates during vegetation green-up [13, 42, 61, 83, 85, 91, 117, 136, 142]. In contrast, kudu (*Traphelagus strepsiceros*) in African savannas decrease daytime activity with ambient temperature—but at different temperature thresholds in dry versus wet seasons, which may vary with pelage thickness [111, 112]. Many mammalian herbivores also alter diet choice, foraging selectivity, and space use across seasons [1, 4, 33, 77, 111, 129]. In sum, abundant evidence exists that mobile terrestrial species exhibit different movement behavior in different seasons. It is thus critical to explicitly account for these seasonal movement dynamics—or risk confounding spatial, temporal, and environmental sources of variation, leading to inaccurate inferences and inappropriate recommendations for conservation and management [21]. One approach to account for seasonal variation is to delineate time periods within which animal movement behavior is relatively consistent.

Many previous studies have defined seasonal periods using date ranges or expert knowledge [13, 67]. However, these approaches may produce relatively arbitrary seasons influenced by individual and geographic bias [63], and may be particularly inaccurate in landscapes with high inter-annual variability. Osipova et al. [109] hypothesized that dry season conditions in the Greater Amboseli Ecosystem constrained elephants (*Loxodonta africana*) to a small number of resource patches. However, only models that used long-term vegetation measurements to define seasons accurately captured connectivity during wet, dry, and transitional seasons, revealing the strong effects of seasonality on functional connectivity for elephants moving through this landscape [109]. As in this example, inappropriately aggregating seasons may inflate variation in movement behaviors and lead to failure to detect ecologically meaningful differences between seasons (i.e., Type II errors). Masking seasonal variation may also have other consequences, such as failing to identify important resources or habitats during key reproductive or developmental periods.

An alternative approach is to define seasons based on environmental conditions, such as snow depth or cover [17, 44, 71], photoperiod [124], or plant phenology [19, 87, 116, 143]. In highly seasonal systems—i.e., where seasonal changes in environmental conditions are of a relatively large magnitude and/or occur relatively rapidly over time—we expect mobile animal species to perceive and respond to cues, thresholds, or other meaningful changes in environmental conditions. However, environmental conditions often vary over fine spatial and/or temporal scales, which presents challenges for identifying both the threshold(s) relevant for a particular species, and appropriate methods to discretize environmental variation into distinct seasons. Moreover, even multiple environmental proxies are unlikely to capture all the factors that influence mobile animal species, because animal behavior integrates over many external variables and internal conditions [71, 98]. For example, although multiple authors investigated the habitat preferences of calving moose cows (*Alces alces*) in Canada, results were inconsistent and conflicting across studies [84]. Lemke [82] delineated four seasons based on a literature review of moose habitat preferences, while Gillingham and Parker [56] used the species’ biology and regional environmental factors to identify five seasons, and Scheideman [123] and Chisholm et al. [30] developed separate modifications of these five seasons. Variation in the environmental factors and thresholds used to separate “calving” from other seasons by different studies ultimately obscured the vital importance of wetlands and water features for calving cows [46].

Another alternative is to use animal movement or activity patterns to define seasons [9, 18, 122, 136, 137]. Rates and frequencies of animal movements are observable biological phenomena directly related to animal use of the environment. For species that migrate or hibernate, sudden changes in movement at the start and end of these phases can be used to define seasons [5, 25, 34, 99]. Delineating seasons for non-migratory and non-hibernating species is more complex, because movement rates and other behavioral responses to changes in resource availability and environmental conditions may vary substantially across individuals and spatial scales [96].

A growing number of studies have used movement ecology approaches to address this challenge, analyzing tracking data to detect transitions in movement behavior, and using these transitions to define seasons. Ferguson and Elkie [42] delineated seasons for caribou (*Rangifer tarandus*) based on inflection points where movement rate changed sign in a polynomial regression. Vander Wal and Rodgers [137] plotted cumulative distance moved by moose over time, and used a curve-fitting technique and inflection points to identify seasons. Birkett et al. [18] modeled the movement speeds of elephants using piecewise linear regression, and interpreted major breakpoints as transitions between seasons. Van Beest et al. [136] modeled Manitoban elk (*Cervus elaphus manitobensis*) and white-tailed deer (*Odocoileus virginianus*) movement rates using generalized additive mixed models (GAMMs), and interpreted days with significant changes in movement rate as seasonal transitions.

These approaches focus on empirical measurements of behavioral responses by a study species, instead of making a priori assumptions about the timing of seasonal transitions, influence of specific environmental variables, or effects of fine spatial and temporal variation [137]. Empirical approaches also reduce the potential impacts of overly narrow environmental information, geographic bias, or individual preconceptions. Moreover, empirical approaches are replicable, enabling researchers and managers to compare findings across species, landscapes, and time periods. Finally, these approaches may be increasingly appropriate under global climate change as, e.g., seasonal definitions based on historical climate patterns may not reflect current seasonal dynamics [141].

These lessons are particularly important for the scimitar-horned oryx (*Oryx dammah*; hereafter “oryx”), a large dryland ungulate native to Central, North, and West Africa. Prior to their extinction in the wild [40], oryx occupied the sub-desert grasslands of the Sahel, which are characterized by extreme seasonal and interannual variation [10, 54, 100]. Historical observations suggest oryx moved seasonally to track rainfall and vegetation quality: groups of oryx may have moved South in response to early rainfall (ca. late May or early June), North contemporaneous with vegetation green-up (ca. July), dispersed in response to widespread vegetation senescence (ca. January), and South again to escape increasing heat and aridity (ca. March [100–102, 106]). Since 2016, more than 300 oryx have been released into the Réserve de Faune de Ouadi Rimé-Ouadi Achim (RFOROA), a large protected area in Chad, through a reintroduction effort led by the Environment Agency—Abu Dhabi (EAD), the government of Chad, and Sahara Conservation [31].

We used an empirical approach to delineate seasons for oryx reintroduced into the RFOROA. More specifically, we compiled tracking data collected by GPS / satellite collars worn by reintroduced oryx, fit GAMMs to mean daytime hourly movement rates across a generic calendar year, and identified seasonal transition dates at the population level. We expected oryx movement behavior to vary over the year, with lower daytime movement rates during hot or dry periods, and higher rates during cool or rainy periods [48, 60]. We also expected that reproductive state would affect the movement behavior of individual oryx—especially for females accompanied by young calves, which exhibit reduced movement capacity for several weeks after birth. Finally, we also sought to evaluate the extent to which captive-born oryx exposed to an arid, highly seasonal environment responded to seasonal cues, as a broad assessment of pre-release practices and other reintroduction protocols. Defining ecologically meaningful seasons for reintroduced oryx will facilitate future analyses, for example to detect the potential re-emergence of seasonal movements, and to assess seasonal variation in habitat selection, diet choice, and space requirements. This information is also essential for managing the reintroduced oryx population and the broader reserve, especially to identify priority periods for monitoring, and sites or periods for enhanced enforcement or potential restrictions on human use of the RFOROA.

## Methods

### Study area

The Réserve de Faune de Ouadi Rimé-Ouadi Achim (RFOROA) is a large (≥ 75,000 km^2^) protected area in central Chad that was gazetted in 1969 to protect scimitar-horned oryx, addax (*Addax nasomaculatus*), dama gazelle (*Nanger dama*), Dorcas gazelle (*Gazella dorcas*), West African cheetah (*Acinonyx jubatus*), African wild dog (*Lycaon pictus*), and other species of regional concern [100]. The reserve contains characteristic Sahelian land cover types including large expanses of sandy soils, sparsely vegetated seasonal grasslands, open savannas, rock outcrops, *ouadis* (sandy streambeds where water is present during the rainy season) surrounded by trees and shrubs, and ephemeral wetlands that support migrating and overwintering bird species [47, 104, 107]. A strong North–South rainfall gradient results in an annual accumulation of ca. 20 mm precipitation near the reserve’s Northern boundary and ca. 400 mm near its Southern boundary.

The movements of the Intertropical Convergence Zone produce a single, short rainy season during June–October, with most rain falling in brief, relatively small, and unpredictable storms in July and August. Various authors have described three, four, or five seasons for the region—including a short rainy season, a growing season, a transitional season, a cool, dry season, and a hot, dry season—but all agree on its extremely strong seasonality and high inter-annual variability [10, 54, 100]. In general, temperatures range from 5 °C in January to 40–50 °C in April, with typical daily temperature ranges of 20 °C and a mean annual temperature near 30 °C.

Hunting is prohibited in the RFOROA, and this prohibition is enforced by the Direction de la Faune et des Aires Protégées within the Ministère de l’Environnement de l’Eau, de la Pêche et du Développement Durable. Agro-pastoralists travel extensively across the reserve, grazing hundreds of thousands of livestock (primarily camels, cattle, sheep, and goats), accessing water, and practicing small-scale agriculture [100, 139]. Multiple aid and development campaigns have established borehole wells (where water is drawn by mechanized pumps), other wells (where water is drawn by hand or livestock), and *hafiris* (natural depressions manually enlarged to increase capacity for, and extend the availability of, surface water) throughout the RFOROA to facilitate human access to potable water [66, 76, 110].

### Study species

Oryx are large (120–200 kg) antelope with cream and rufous coloring and long (80–150 cm), ridged horns that arch over their back [16, 51, 94]. The species was formerly abundant across the semi-arid grasslands fringing the Sahara Desert, with historical occurrence records from Egypt, Libya, Tunisia, Algeria, Morocco, Mauritania, Mali, Niger, Senegal, Burkina Faso, Nigeria, Chad and Sudan [16, 78]. Oryx are predominantly grazers, and have been observed to consume a wide variety of grasses including *Panicum turgidum*, *Panicum laetum*, and *Aristida mutabilis*; they will also consume leaves of shrubs such as *Chrozophora senegalensis* and *Indigofera* species, as well as other plants including the wild desert melon *Citrullus colocynthis* [10, 36, 37, 53, 54, 100]. A combination of overhunting, political conflict, and increasing competition with domestic livestock led to the species’ decline across its range [22, 23, 39, 78, 103, 105]. The last confirmed sightings of oryx in the wild occurred in Chad in the 1980s [89, 106], the species was classified as Extinct in the Wild in 1999 [40] and recently reclassified as Endangered, due to ongoing reintroduction and conservation efforts (IUCN SSC Antelope Specialist Group 2023).

Throughout their decline in the wild, oryx were maintained in captive collections, partially due to their importance in North African and Middle Eastern cultures [20, 55, 62, 106]. From an estimated 40–45 founders representing multiple lineages, the current global captive population now comprises 15,000–20,000 individuals at more than 400 institutions across nearly 50 countries [50, 68, 144]. Oryx were released into Bou Hedma National Park, Tunisia in 1985 [48] [15, 59, 60, 74]), Souss Massa National Park, Morocco during 1995–1997 [97], Sidi Toui National Park and Oued Dekouk National Reserve, Tunisia and Guembeul Faunal Reserve, Senegal in 1999 (Wakefield and Molcanova 2001 [92]),the Réserve de Faune du Ferlo Nord, Senegal in 2003 [69, 70], and Dghoumes National Park, Tunisia in 2007 [52]. Due to the small size of these reintroductions (5–25 animals), their restriction to fenced enclosures, and concerns about long-term population viability, conservation planning workshops were convened during 2009–2012 to explore opportunities to reintroduce oryx into larger, unfenced protected areas [14, 80, 81].

In 2013, the Environment Agency—Abu Dhabi (EAD), the government of Chad, and Sahara Conservation initiated the reintroduction of oryx into the RFOROA. All founder animals originated from the “World Herd” managed by EAD, were translocated to the reserve by air and road, and were held in 20-ha enclosures for 1–6 months. Before release, oryx were restrained in a drop-chute device (Fauna TAMER Jr, Fauna Research Inc., Red Hook, NY, USA) for less than 10 min, during which time a brief veterinary exam was performed, biological samples were collected, and a GPS / satellite collar was fit to most (> 95%) individuals. Animal handling methods were authorized under a cooperative agreement between Sahara Conservation and the government of Chad, and approved by the Animal Care and Use Committee of the Smithsonian’s National Zoo and Conservation Biology Institute. Since August 2016, more than 300 oryx have been released into the reserve.

### Animal movement data

**Fig. 1:**
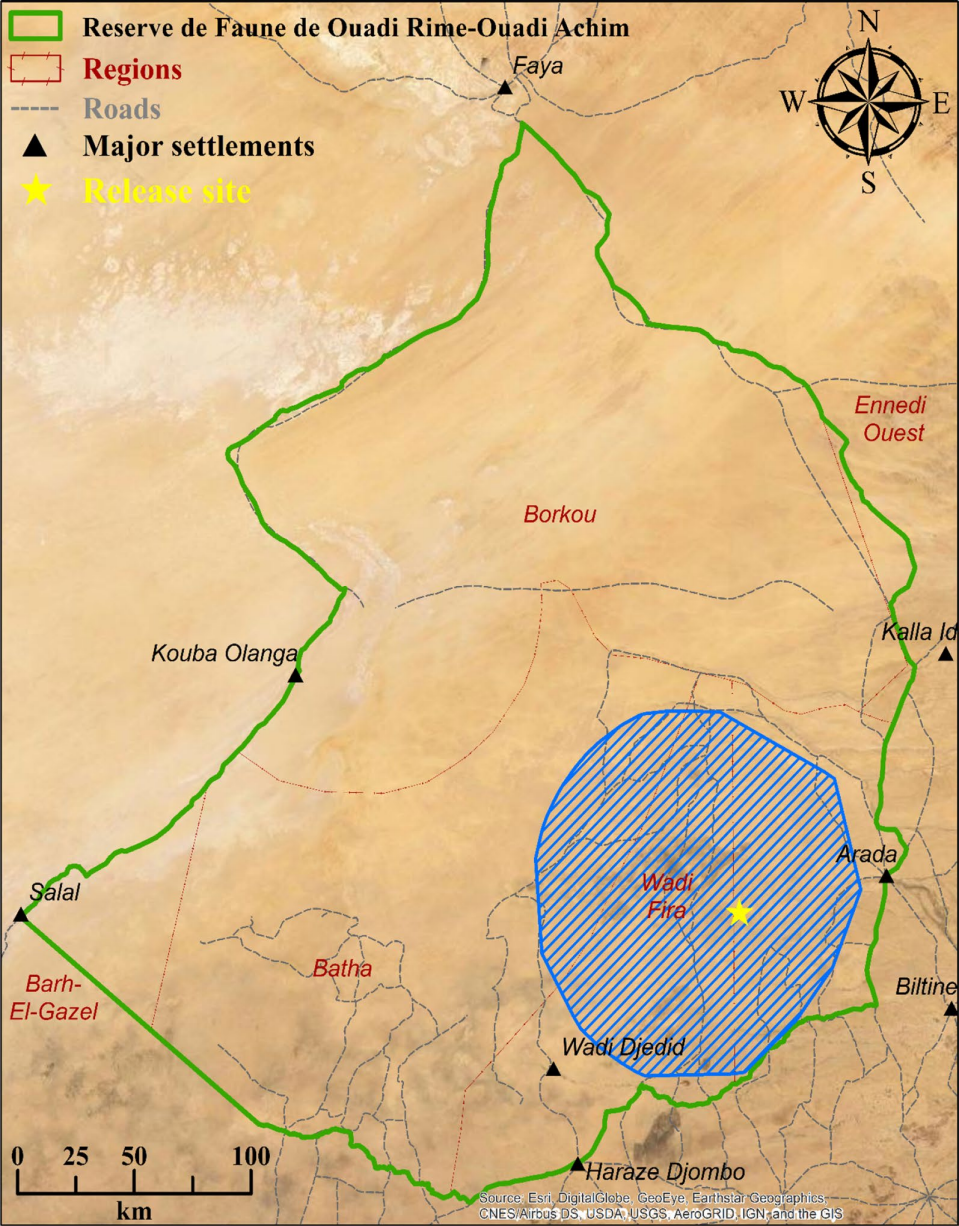
Map of the Réserve de Faune de Ouadi Rimé-Ouadi Achim, Chad. The area *highlighted in blue* shows the 95% minimum convex hull for oryx movement data collected during 2016–2022, and indicates where reintroduced oryx are typically found within the reserve

We compiled movement data collected by GPS / satellite collars fit to oryx reintroduced into the RFOROA through 2022 (Fig. 1). We removed positions that indicated GPS fix or timestamp errors, DOP less than 5.0, or biologically unrealistic movement rates (> 18 km/h). We excluded the first 10 weeks after release from further analysis, as reintroduced animals may display erratic or atypical movements during this period [65, 90, 118, 125], and extensive exploratory movements immediately after release have been documented in this population [86]. Finally, we also removed positions within two weeks of known mortalities to exclude the effects of disease or other causes of death. All data preparation and analysis steps were implemented in R [115].

**Fig. 2:**
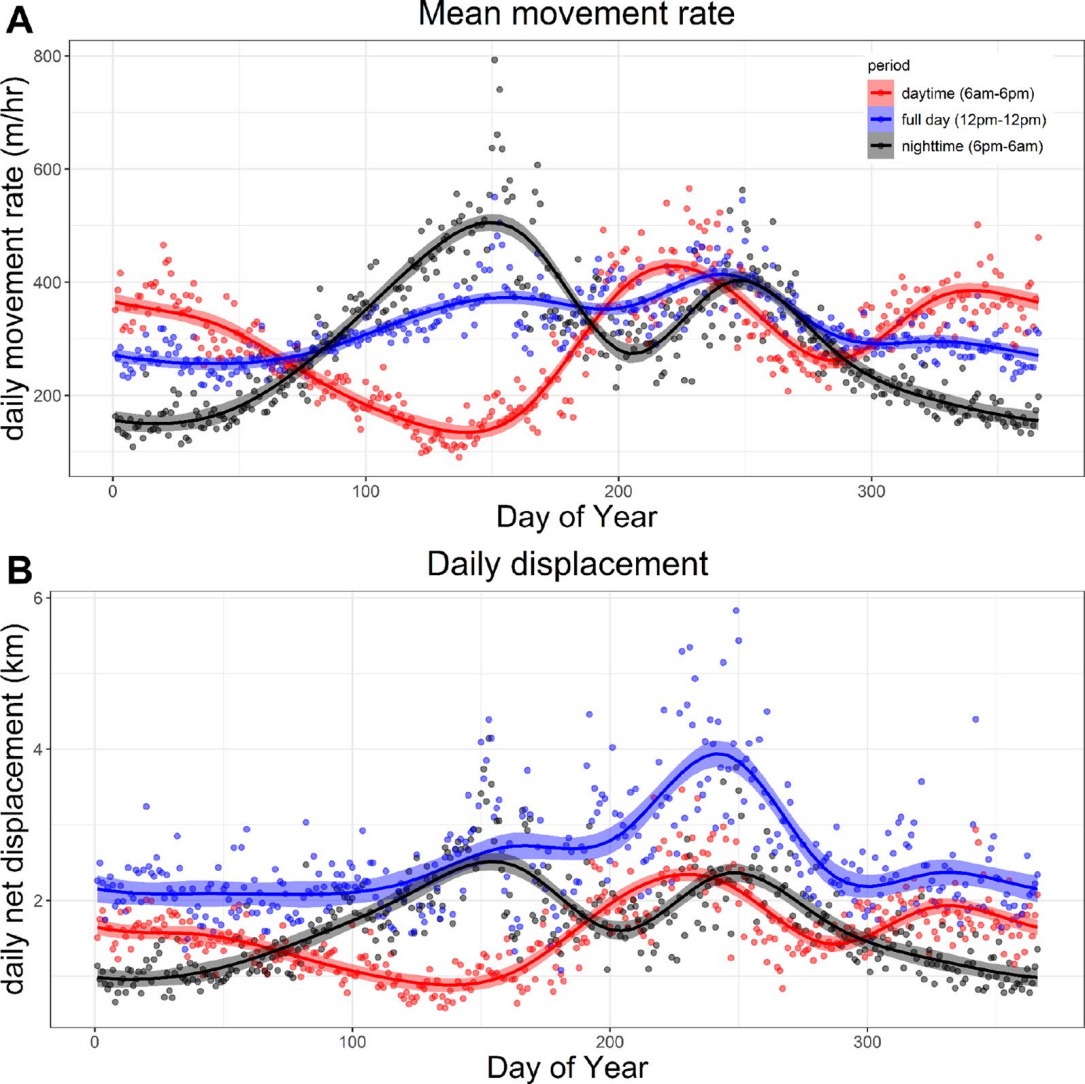
**A** Hourly movement rates and **B** Daily net displacement by reintroduced scimitar-horned oryx across the calendar year. Movement metrics were calculated using tracking data from 104 individuals and 2048 unique dates during 2016–2022, over 24-h days (*blue*), daytime periods (ca. 6 am-6 pm, *red*), and nighttime periods (ca. 6 pm-6am, *black*). Points indicate mean values across all individuals on a given day, lines indicate trends smoothed using a thin plate spline approach, and shaded areas show standard error around smoothed values

From the resulting movement data, we calculated mean daytime hourly movement rates for each individual. Because mean daytime (06:01–18:00) temperatures in this region exhibit greater variation over the year than mean nighttime (18:01–06:00) temperatures, we expected daytime movements by reintroduced oryx to respond to this variation. Indeed, daytime movement rates by reintroduced oryx exhibited greater variation across the year compared to nighttime and full-day (00:00–23:59) movements (Fig. 2). We calculated mean daytime hourly movement rates by dividing the distances between all consecutive pairs of retained positions within the daytime period by the difference between their start and end times, then took the mean of these values. To avoid biases towards specific collar fix schedules (1 h, 2 h, or 4 h interval between GPS fixes) or times of day, we calculated the mean daytime hourly movement rate for an individual oryx only on days when (1) at least three high-quality GPS positions or 50% of expected steps were available, and (2) these positions extended over at least 50% of the daytime period (i.e., at least 6 h between 06:01 and 18:00). To reduce bias towards specific individuals and times of year, we only included oryx for which movement data were available on at least 50% of the days of the year (DOYs) between Jan 1 and June 30, and at least 50% of the DOYs between July 1 and Dec 31. The resulting data set contained 57,255 mean daytime hourly movement rates from 104 oryx (38 males and 66 females) over 2048 unique days from 2016 to 2022, and contained 210–1670 movement rates per oryx.

For each day when a mean daytime hourly movement rate could be calculated for an individual oryx, we determined its age and reproductive state, as these factors have previously been shown to influence animal movement [136]. Age was extracted from EAD birth records and ranged from 1.4 to 9.1 years. All males were assigned a reproductive status of “male”. Female reproductive status incorporated information about an animal’s current pregnancy and current calf. Female oryx typically conceive their first offspring at 27.8 months (range: 19.1–46.7 months), gestate for 258 days, and exhibit an interbirth interval of 277 days (range: 241–705 days [24, 35, 51, 57, 93]). Ovulation typically occurs soon after parturition, such that most conceptions occur within three months of a previous birth [93]. We thus defined three possible pregnancy states: “early term” (estimated conception date through 129 days after conception), “late term” (130 days after conception through the day before birth), and “not pregnant.”

We extracted calf birth dates from direct observations conducted daily by a monitoring team based in the RFOROA. Most calves were detected within 1–2 days of birth; otherwise, horn morphology, the ratio of horn length to head length, and pelage were used to estimate calf birth dates. We estimated the conception date for a given calf by subtracting 258 days from its birth date. (Where this calculation indicated conception before the known birth date of a previous calf, the conception date was modified to the day after the previous calf’s birth date). Any day ≥ 258 days after a known birth, or when birth details were uncertain, received a pregnancy state of “unknown” and was excluded from further analysis.

We developed calf states based on historical observations of oryx before extinction in the wild and modern observations of oryx in captivity. Pregnant oryx, either individually or in small groups, often separate from social groups shortly before calving and rejoin them 3–14 days after giving birth [58]. Oryx calves conceal themselves in vegetation at some distance from their mothers for 2–4 weeks after birth (termed “hiding” or “tucking” behavior [57]). In captivity, calves may not be weaned until 5–10 months after birth, however in the wild most oryx calves are weaned around four months after birth [57, 101, 102]. We used this information on typical behavior by oryx dams and calves to define three possible calf states: “neonate at heel” (estimated calf birth date within one month, calf performing largely hiding behaviors); “calf at heel” (estimated calf birth date within 1–4 months, calf largely following dam); and “no calf” (no known calf birth date or mortality within the previous four months). The reproductive state for a female oryx on any given day was the combination of its pregnancy and calf states (n = 7 possible states).

### Generalized additive mixed model structure and evaluation

We used generalized additive mixed models (GAMMs) implemented in a Bayesian framework to predict mean daytime hourly movement rates by reintroduced oryx across the calendar year. To understand how oryx perceive and respond to seasonal changes, we included day of year (DOY; 1–366) as an explanatory variable in all models. We then developed candidate models that represented competing hypotheses about factors that influence oryx movement rates: age, reproductive state, and release conditions (release group 1–6). DOY and age were centered and scaled, and the response variable of mean daytime hourly movement rate was log-transformed. All but the simplest candidate model contained an autoregressive moving average term to account for temporal dependence among consecutive movement rates. Based on inspection of partial autocorrelation functions, the median lag by which temporal autocorrelation was sufficiently reduced across all oryx was 5 days. We therefore used individual days as the time variable, individual identity as the grouping variable, an autoregressive order (*p*) of 5, and a moving average order (*q*) of 0. All but the simplest model also included a random intercept for individual identity (see Table S1 for a full list of candidate model structures).

In all models, we used the gaussian distribution family, set DOY as a cyclic cubic regression spline smooth (requiring the end of the spline on Dec 31 to smoothly meet its beginning on Jan 1), and set age as a thin plate spline smooth. We confirmed that our data met the assumptions of a gaussian distribution through visual inspection of Q–Q plots, plots of residuals vs. fitted values, and plots of residuals vs. linear predictors. We used only ‘by’ smooths to represent the interactions between reproductive state and DOY, and reproductive state and age, to allow different smoothness parameters for each reproductive state. We set the *k* of each smooth (a parameter governing the ‘wiggliness’ of the response curve) to an intermediate value of 10. We ran each GAMM for 4 chains of 10,000 iterations, setting aside the first 4000 for warmup. Adapt delta and max treedepth were set to 0.99 and 20, respectively. Models were implemented in the *brms* package [26] through its interface with Stan [27], and run on the Hydra high-performance computing cluster [128].

We assessed model convergence by examining R-hat (Gelman-Rubin statistic) values for all parameters. We evaluated model fit by examining posterior predictive check plots, including density overlay plots and mean predictions from simulated data. We selected a final model based on fit, variance explained, and leave-one-out cross-validation (a Bayesian estimate of out-of-sample predictive accuracy similar to AIC) calculated using the *loo* package [138].

### Delineating seasons

**Fig. 3:**
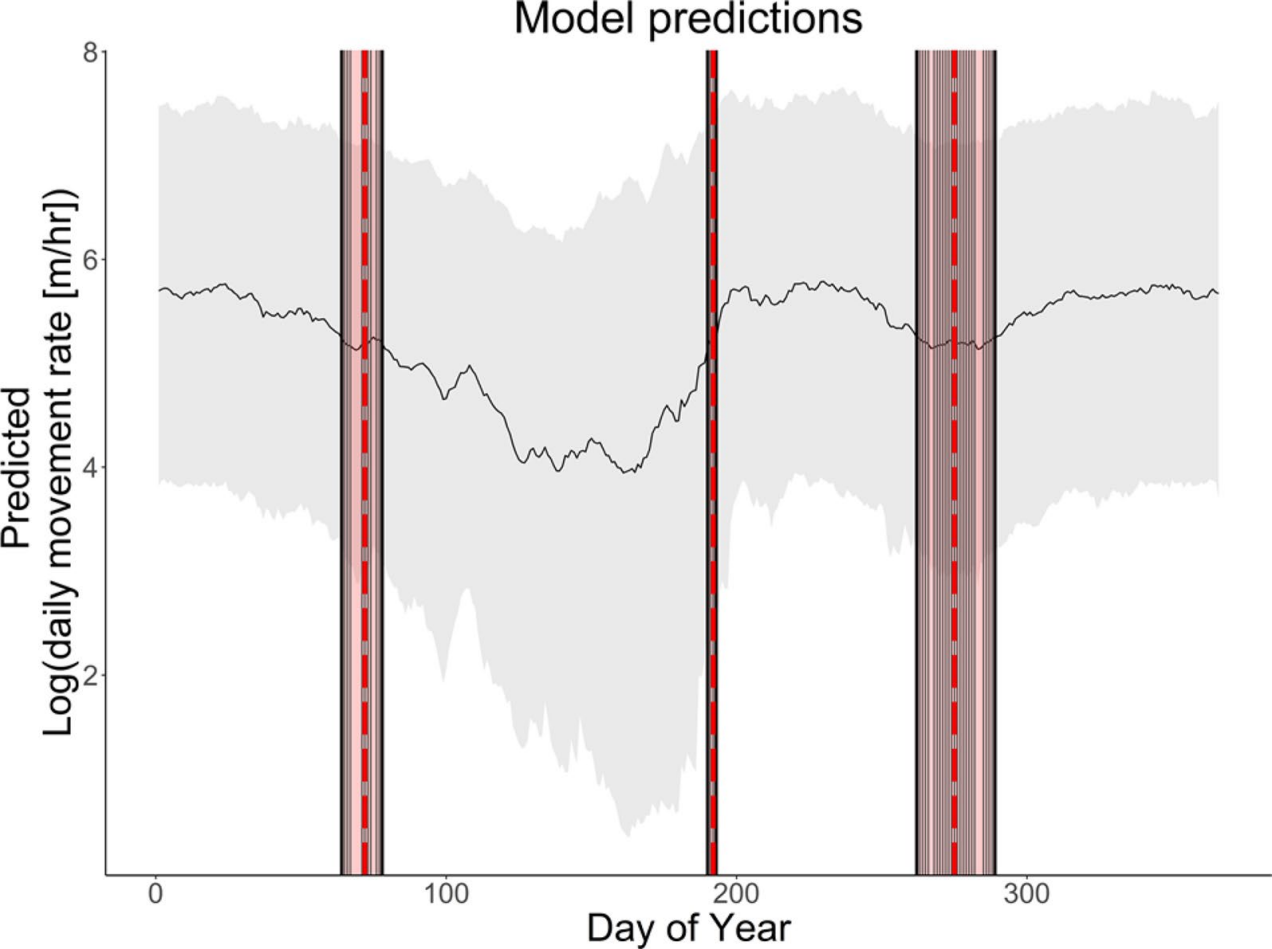
Predicted movement rates and dates of seasonal transitions for reintroduced oryx. *Black line* shows log-transformed, population-level mean daytime hourly movement rates across the calendar year, as predicted by the final model; *gray shading* shows upper and lower 95% confidence intervals around model predictions. *Vertical black lines* indicate each day of the year (DOY) when the predicted population-level movement rate passes through the global mean, while *red shading* shows the three brief periods during the calendar year when these transitions occur. *Vertical dashed red lines* indicate the median DOY of each period, which represent seasonal transitions in movement behavior

We used the *posterior_predict* function from the *brms* package to calculate the global mean of all mean daytime hourly movement rates predicted by the final model. We considered periods when the predicted population-level mean daytime movement rate passed through this global mean, persistently increased or decreased towards a limit, then gradually returned to the global mean, to represent bouts of relatively consistent movement behavior. During three short periods across the calendar year, the predicted population-level mean daytime hourly movement rate transitioned through the global mean (Fig. 3). We used the median DOY from each of these transitional periods to capture start and end dates for candidate seasons. To assess how effectively this approach delineated ecologically meaningful seasons for reintroduced oryx, we tested quantitative associations between movement rates predicted by the final model and environmental conditions considered critical in arid landscapes. Specifically, we calculated Pearsons’s R correlation between predicted daytime movement rates and mean daily daytime temperature and relative humidity (as measured by a meteorological station near the release site) within each candidate season.

## Results

Our final data set included 57,255 mean daytime hourly movement rates from 104 oryx released into the RFOROA between 2016 and 2021 (mean days of data per oryx = 581). The mean daytime hourly movement rate across the final data set was 300 m/h (SE = 1 m/h).

### Population-level results

Our final model included a smooth for DOY by reproductive state, a smooth for age by reproductive state, reproductive state, and release group as predictor variables; individual identity and year as random effects; and an autoregressive structure to capture temporal autocorrelation. (See Table S1 for all candidate model structures). The final model explained over half the variation in oryx movement rates across the year, achieved the highest Bayesian R^2^ of all candidate models (Bayesian R^2^ = 0.56), and reported the lowest looic across all models containing a smooth for DOY by reproductive state. No R-hat values exceeded one, indicating adequate chain mixing and model convergence, and model diagnostic and posterior predictive check plots indicated overall good predictive performance (Figure S1). Population-level predictions from our final model indicate that oryx movement rates vary substantially across the calendar year, with three periods when the predicted mean daytime hourly movement rate transitions through, then remains persistently higher or lower than, the global mean (Fig. 3).

Based on these transition dates, meteorological data collected near the release site, and regional season naming conventions, the “hot, dry” season begins on March 13 and lasts through July 10 (120 days). During this period, rain has not fallen since the previous year (at least five months prior to the season's start date) and daytime temperatures often exceed 40 °C. Daytime movements by reintroduced oryx reach annual lows during this season, falling to a minimum predicted hourly movement rate of 52 m/h (on DOY 161, June 10) and a mean of 93 m/h. The hot, dry season is followed by the “rainy” season, which starts on July 11 and ends on October 1 (83 days). The rainy season contains the peak mean daytime hourly movement rates predicted for reintroduced oryx: a maximum of 327 m/h (on DOY 230, August 18) and mean of 255 m/h. The “cool, dry” season encompasses the remainder of the year, beginning on October 2 and lasting through March 12 (162–163 days), after the annual rains and when temperatures typically remain relatively low. During this season period, reintroduced oryx exhibit similar movement behaviors to the rainy season, but reach a slightly lower maximum daytime movement rate of 317 m/h (on DOY 24, January 24) and mean of 252 m/h.

**Fig. 4:**
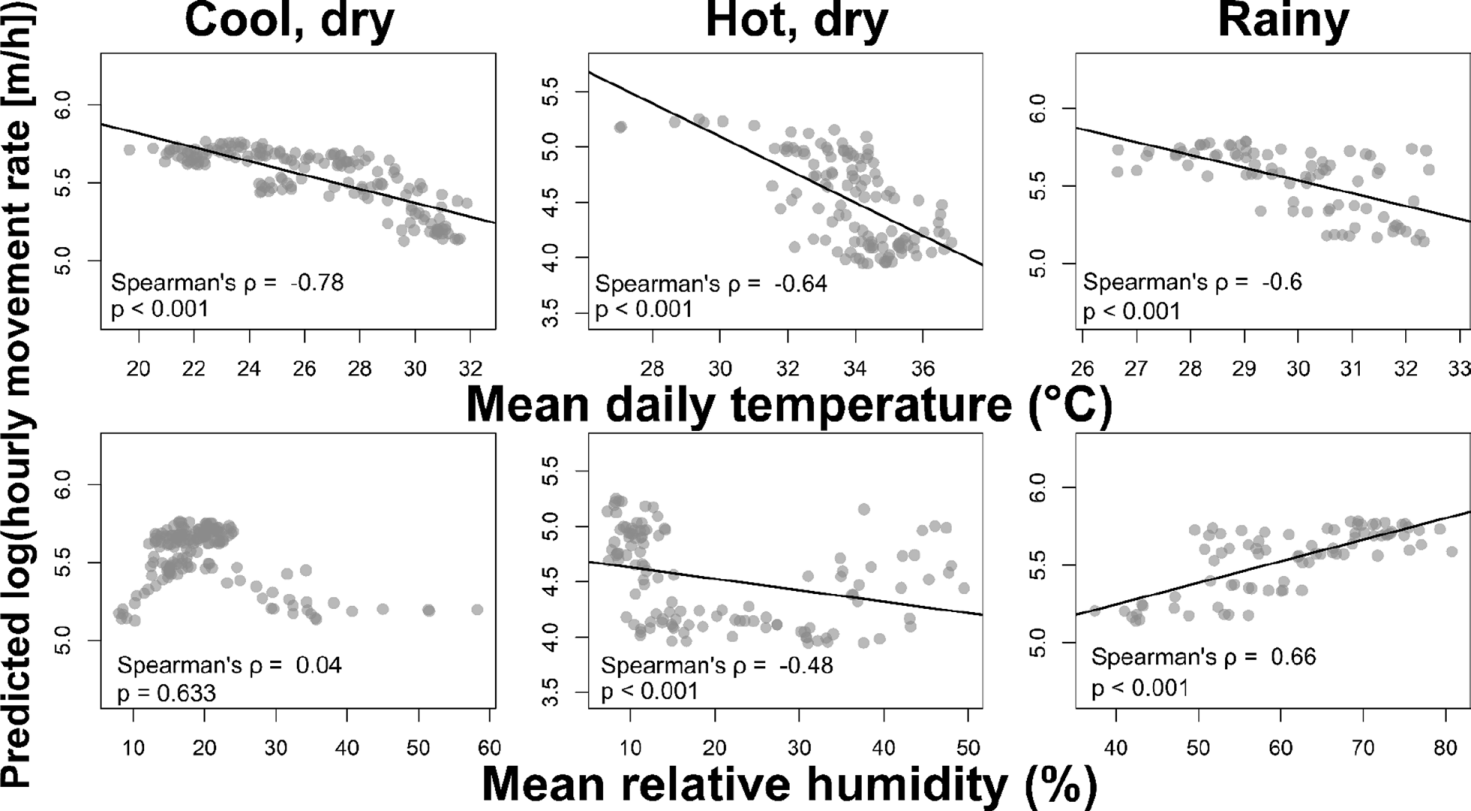
Predicted movement rates are correlated with local meteorological variables within delineated seasons. Population-level mean daytime hourly movement rates predicted by the final model were strongly and significantly correlated with two environmental variables—daytime temperature and humidity—often considered critical in arid landscapes. These associations provide complementary support for the seasonal delineations indicated by our final model

These three movement-defined seasons align well with the annual environmental variation characteristic of southern Sahelian landscapes. Population-level mean daytime hourly movement rates predicted by the final GAMM were significantly negatively correlated (Pearson’s R, *p* < 0.001) with mean daily daytime temperature for all delineated seasons (Fig. 4); this relationship was strongest during the cool, dry season. Predicted movement rates were also significantly negatively correlated (Pearson’s R, *p* < 0.001) with mean relative humidity during the hot, dry season, and significantly positively correlated with humidity during the rainy season (Pearson’s R, *p* < 0.001); this relationship was not significant during the cool, dry season.

### Influences of model covariates

**Fig. 5:**
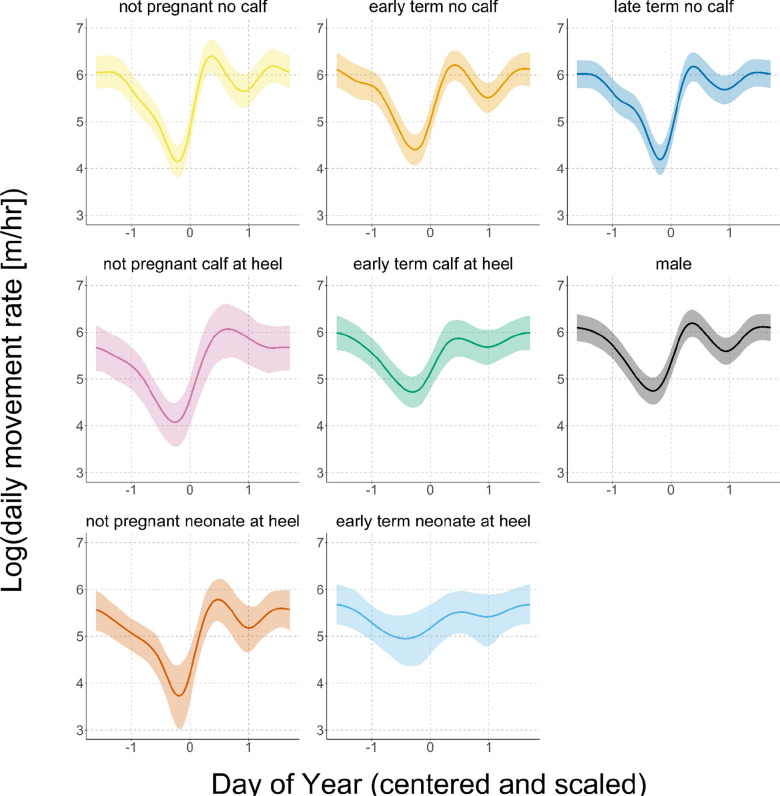
Reproductive state affects predicted movement rates for reintroduced oryx. Individual panels show the conditional effects for each level of the smooth term for day of year by reproductive state from the final model. *Colored lines* show the predicted mean daytime hourly movement rate over the calendar year for each reproductive state, and *colored shading* indicates the 95% confidence interval around model predictions

All levels of the smooth term for DOY x reproductive state exhibited an overall pattern of decreasing to an annual nadir during the hot, dry season, and increasing to a peak during the rainy season (Fig. 5). All model coefficient estimates of smooth terms for DOY x reproductive state were significant (no 95% confidence intervals overlapped 0), indicating that this variable impacts the magnitude and timing of changes in movement rates by reintroduced oryx. Males exhibited the greatest predicted movement rates overall, followed by female oryx with no calf at heel. Females in the late stages of pregnancy (“late term”) and females that were not pregnant exhibited the greatest variation in movement rates across seasons, while females in the early stages of pregnancy (“early term”)—particularly early term females with a neonate at heel—exhibited the least variable movement rates across the calendar year. Not pregnant females with neonates at heel achieved the lowest population-level estimated movement rate of 42 m/h (SE = 1 m/h), and all not pregnant females exhibited lower nadirs during the hot, dry season, and higher peaks during the rainy season, compared to early term females of the same calf state.

While all reproductive states increased movement rates at the beginning of the rainy season, early term females with neonates at heel and not pregnant females with calves at heel exhibited more gradual increases, reaching peak movement rates on DOY 237 and 248, respectively. In addition, estimated coefficients for the reproductive states not pregnant with neonate at heel (− 0.53 ± 0.10, 95% CI − 0.73 to − 0.32), not pregnant with calf at heel (− 0.35 ± 0.11, 95% CI − 0.57 to − 0.15), and early term with neonate at heel (− 0.30 ± 0.11, 95% CI − 0.53 to − 0.07) were also significant in the final model.

**Fig. 6:**
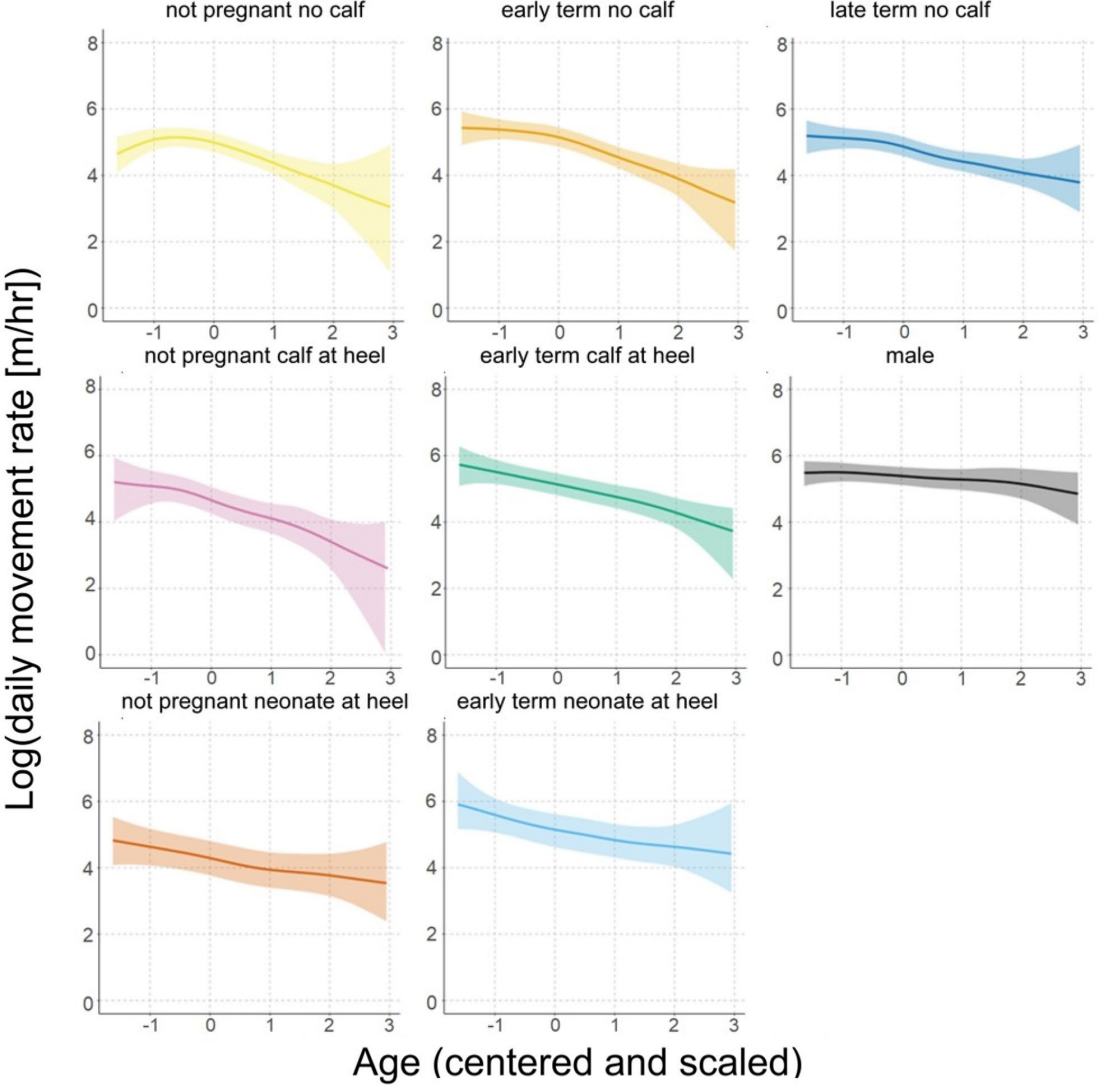
Age weakly and negatively influences predicted movement rates in reintroduced oryx. Individual panels show the conditional effects for each level of the smooth term for age by reproductive state from the final model. *Colored lines* show the predicted mean daytime hourly movement rate across different ages for each reproductive state, and *colored shading* indicates the 95% confidence interval around model predictions

Similar to DOY by reproductive state, all levels of the smooth term for age by reproductive state were significant (no 95% CIs overlapped 0). Overall, predicted movement rates exhibited a gradual reduction with age for all reproductive states—with the exception of not pregnant females without calves, which exhibited a small initial increase in movement rates, peaking at 3.1 years of age before gradually declining (Fig. 6). Males exhibited the smallest decrease in predicted movement rates with age, while not pregnant females with calves exhibited the largest decrease.

**Fig. 7:**
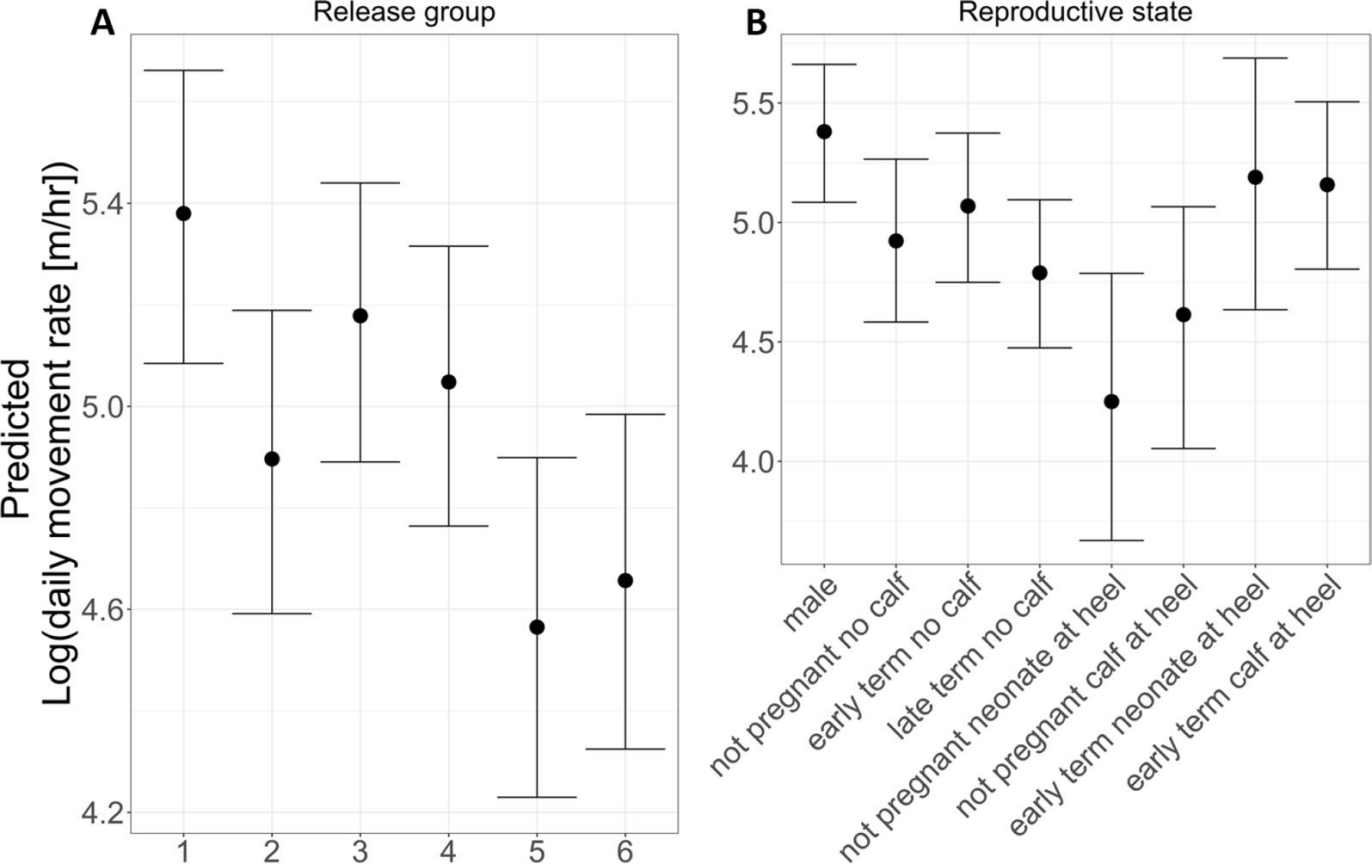
Impacts of release conditions and reproductive state on movement rates by reintroduced oryx. **A** The conditional effects of the categorical variable for Release Group included in the final model indicate increasingly negative impacts on daytime movement rates across successive releases. The release group variable aggregates multiple factors that may affect oryx movement, including length of acclimation period, forage (and other environmental) conditions at release, and the number of oryx already present in the reserve. **B** Conditional effects across reproductive states indicate that male, early term, and not pregnant, no calf reproductive states have the strongest positive impact on daytime movement rates, while late term and not pregnant reproductive states have decreased impacts. Plots are based on another categorical factor as reference level (male reproductive state for A, release group 1 for B)

The categorical predictor variable “release group”—which aggregates multiple factors including number of animals released, length of acclimation period, environmental conditions at release, and the number of oryx present in the reserve at release—also influenced predicted movement rates (Fig. 7). The coefficient representing Release 1 was positive and highly significant (5.70 ± 0.12, 95% CI 5.47–5.94), while estimated coefficients for all other release groups were negative, smaller, and predominantly significant, except for Release 3. Across all release groups, Release 5 (− 0.81 ± 0.17, 95% CI − 1.14 to − 0.48) and Release 6 (− 0.72 ± 0.17, 95% CI − 1.05 to − 0.39) exhibited the lowest predicted movement rates.

The random effects of year (0.07 ± 0.05; 95% CI 0.01–0.18; see Figure S2) and individual identity (0.39 ± 0.03; 95% CI 0.33–0.46; see Figure S3) also impacted predicted movement rates. Most posterior estimates of random effects for individual oryx overlapped zero; however, select animals exhibited significantly negative (e.g., 39853.1, 29917, 22113.2, 22110.2, 22109.2, and 21398) or positive (e.g., 32040.2 and 30170.1) deviation (Figure S3). The estimated random effect of year was barely significant (0.07 ± 0.05; 95% CI 0.01–0.18), and all 95% credible intervals of posterior estimates for individual years overlapped zero, indicating minimal influence of year compared to other covariates. All sequential autocorrelation structures were significant (no 95% CIs overlapped 0), and decreased sharply from 0.28 (± 0.00; 95% CI 0.27–0.29) at lag = 1 to 0.09 (± 0.00; 95% CI − 0.08 to 0.08) at lag = 5.

## Discussion

Movements by scimitar-horned oryx reintroduced into the Réserve de Faune de Ouadi Rimé-Ouadi Achim (RFOROA) in Chad vary substantially over the calendar year (Fig. 2). Reintroduced oryx dramatically decrease daytime movement rates during the hottest months of the year, and increase daytime movements when high-quality food resources (i.e., growing grasses) are abundant. Our final generalized additive mixed model (GAMM), built using mean daytime hourly movement rates across more than 6 years and 100 animals, indicated that oryx movement rates exhibit three transitions per year. Within the periods delineated by these transitions, movement rates persistently increased or decreased toward a mid-period limit, then gradually returned to the global mean (Fig. 3). Predicted mean daytime movement rates within periods were significantly correlated with environmental variables widely considered to define and constrain arid systems. However, these relationships varied among periods, such that mean daytime hourly movement rates were either positively, negatively, or not significantly correlated with mean relative humidity within individual periods. Together, these results indicate that oryx reintroduced into the RFOROA experience three seasons per year.

Reintroduced oryx exhibited peak daytime movement rates during the rainy season (seasonal maximum = 327 m/h, mean = 255 m/h), likely to maximize access to green and growing herbaceous vegetation. In this season, daytime surface air temperatures are generally warm (ca. 20–35 °C) and localized rainstorms enable the growth of patches of annual and perennial grasses; conditions that represent relatively low energetic costs for extensive movement (e.g., low thermal load and risk of water loss), compared to potential reward (e.g., calorie gain from locating and consuming high-quality forage). During the cool, dry season, reintroduced oryx maintained similar daytime movement rates (seasonal maximum = 317 m/h, mean = 252 m/h), likely reflecting continued low costs for movement as temperatures cool somewhat (to ca. 15–30 °C), and the continued—though diminishing—availability of forage. However, during the hot, dry season, elevated temperatures (ca. 25–40 °C) increase thermal stress, and nutritious vegetation is rare and patchy; under these conditions, the likely benefits of daytime movements fall dramatically, and oryx exhibit the lowest daytime movement rates of the year (seasonal mean = 93 m/h).

Reduced daytime movement rates, and thus likely reduced foraging, by oryx during the hot, dry season may be analogous to winter inappetance among ungulates in arctic and northern temperate zones [6, 132]. Reduced intake during the winter months enables these populations to minimize energy expenditure when the likely benefit to body stores is low [88, 113, 134, 135]. Reintroduced oryx may compensate for reduced intake through decreased metabolic rates, similar to white-tailed deer occupying arid regions of the southern United States [131]. Alternately, oryx also exhibited higher nighttime movement rates during the hot, dry season (see Fig. 2), suggesting a possible behavioral adaptation to maintain minimum intake, potentially to sustain the rumen microbial community [64].

Our final GAMM also revealed substantial variation in predicted daytime movement rates across the year among reproductive states. Late term and not pregnant females exhibited large, rapid declines in daytime movement rates approaching the hot, dry season, low movement rates during the hot, dry season, and high movement rates during the rainy season. Early term females exhibited similarly timed increases and decreases in daytime movement rates; however, the magnitude and slope of these changes were smaller, and movement rates during the hot, dry season were higher, compared to other female reproductive states. Early term females with neonates at heel also exhibited the most elevated, consistent daytime movement rates across all female reproductive states. This limited variation in movement rates over the year for all early term states may suggest that female oryx spend substantial time foraging during early pregnancy, regardless of forage availability.

Producing fetal tissues increases energetic costs, leading to energy demands up to 50% greater in pregnant vs. non-pregnant female ungulates [120, 121]. For pregnant ungulates, the last trimester is generally understood to incur the greatest energy demand, because ca. 80% of fetal mass is produced during this period [120, 121]. Lactation has also been shown to increase energy demand by 65–215% in ungulates, with energetic costs due to lactation considered greatest in females supporting neonates (i.e., calves < 30 days old and somewhat reduced in females supporting older calves (i.e., calves near weaning, which typically occurs after ca. 4 months in reintroduced oryx [28, 32, 108, 114, 119]). Under this paradigm, not pregnant females with neonates at heel, early term females with neonates at heel, and late term females should experience the highest energy demand—and would thus be expected to spend more time foraging to meet these demands, leading to elevated daytime movement rates. However, late-term female oryx exhibited highly variable daytime movement rates over the year, with rates and transition timing most similar to not pregnant females without offspring. Instead, among female reproductive states, early term oryx exhibited the highest daytime movement rates across the year, including during the hot, dry season.

Pregnant and lactating females may draw on recently consumed (i.e., “income”) or stored (i.e., “capital”) resources to meet energy demands, and ungulate species exhibit diverse strategies along the income-to-capital continuum [29, 120]. For example, many ungulates in northern temperate and arctic environments mate in autumn, carry pregnancies through winter when food availability is low, and calve in spring or summer when food availability is increasing (or already high). These species often employ a capital breeding strategy, expending maternal body stores gained during the previous summer and fall to support fetal growth during pregnancy and even early lactation. Capital breeding strategies buffer against unfavorable environmental conditions, a valuable feature in environments with regular cycles in resource availability—and potentially similarly valuable in highly seasonal and unpredictable environments such as the southern Sahel [6, 38, 41].

When faced with limited resources, life history theory [130] and parental investment theory [133] predict that females of large, long-lived species should employ conservative strategies, decreasing the maternal stores allocated towards current reproduction to maintain their own survival and the potential for future reproduction [7, 8, 43, 73]. In Denali National Park, predation by grizzly bears and wolves can remove 87% of caribou neonates each year [2]. After a particularly severe winter, Adams [3] observed that pregnant caribou reduced maternal stores allocated to pregnancy late in gestation, potentially conserving capital to support themselves, or offspring that escape predation. Using income to meet energy demands during early pregnancy, and capital to meet demands during late pregnancy, may represent a conservative strategy for female oryx reintroduced into the RFOROA.

Oryx calves largely hide among vegetation during the first weeks after birth [57, 100]. Post-partum oestrus and ovulation typically also occur during this period, such that most conceptions occurring within three months of birth [59, 93, 101, 102]. Thus, female oryx with offspring may experience multiple, conflicting demands: neonate hiding behavior and the potential need for offspring defense constrain overall movement; the onset of estrus after calving may compel mate-searching behavior; and though oryx may rely somewhat on maternal capital to meet energy demand from lactation, increased foraging may be required to fulfill additional demands incurred by early pregnancy. On most days of the year, not pregnant females without offspring exhibited moderately higher movement rates than not pregnant females with calves, which in turn exhibited slightly higher movement rates than not pregnant females with neonates. However, this pattern was not replicated in early term females—particularly during the hot, dry season, when early term females with neonates exhibited the highest movement rates among female reproductive states. In addition, predicted mean daytime hourly movement rates by male oryx remained relatively high across the year, suggesting that mate-searching movements may be primarily performed by males. Data from routine monitoring of reintroduced oryx in the RFOROA provide anecdotal support for this inference, as male oryx are frequently observed to locate female oryx and recent neonates within hours or days of calving. Together, these outcomes suggest that energy demands during early pregnancy may trump movement constraints from typical neonate behavior.

Differences in daytime movement rates among groups of oryx released into the RFOROA (See Fig. 7) may be used to inform future reintroduction practices. Because the management background unique to oryx released in 2016 (R1) has previously been shown to affect their post-release movements [86], we excluded R1 from consideration. We acknowledge that inferences are limited by our small sample size (n = 5 release groups); however, the large investment required to conduct conservation translocations, the imperative to improve translocation practices, and the likelihood of future ungulate releases in the RFOROA compel an evaluation of potential insights at the release group level—within conservative limits.

After translocation to the reserve, oryx are managed in enclosures to promote acclimation to local conditions before release into the wild. Oryx from release groups that acclimated for at least five months (R3, 6–9 months; R4, 6 months) exhibited somewhat higher movement rates than oryx released after shorter acclimation periods (R2, 2 months; R6, 1 month), with the exception of R5 (7 months). Oryx from groups released early in the rainy season (R3, R4, Aug) also exhibited higher movement rates than oryx released late in the rainy season (R5, Sep) or during the cool, dry season (R2, Jan; R6, Dec). Finally, oryx from larger release groups containing multiple age classes (R3, 37 adults and 14 calves; R4, 73 adults and 3 calves) exhibited higher movement rates than oryx from smaller groups containing only adults (R2, 14 adults; R5, 23 adults; R6, 24 adults). Given these outcomes, managing oryx in relatively large groups comprising multiple age classes, and releasing these groups early in the rainy season, may support increased movement capacity. Mobility was considered a primary characteristic of the species before its extinction in the wild [54, 100–102], and is often proposed as a critical trait for species occupying environments with high spatiotemporal variability [11, 72, 95, 96, 127].

## Conclusions

This study provides further evidence that generic date ranges or environmental proxies are sub-optimal approaches to delineate seasons for mobile animal species. A “rainy season” based on regional conventions or annual precipitation patterns would have ended in late August or early September, inappropriately interrupting the consistent movement behavior exhibited by oryx reintroduced into the RFOROA through October 1. The three ecologically relevant seasons delineated here will facilitate future investigations into the movement ecology of reintroduced oryx, and to inform conservation and management actions. For example, oryx movement data may be partitioned into seasons to investigate variation in habitat preferences and resource selection, which in turn may highlight important sites and resources within the reserve.

Ensuring that animals sourced from captive collections acclimate to in situ environmental conditions is a major challenge in conservation translocations [12, 45, 126]. Releases into environments with strong seasonality or other high spatiotemporal variation is particularly challenging, as translocated animals may encounter different sets of environmental conditions over relatively short time periods, and must not only acclimate to each, but shift among them at appropriate times [75, 79]. That naive oryx, raised in a captive setting where they did not perform natural foraging or resource tracking behaviors, exhibited transitions in movement behavior aligned with the seasonal variation characteristic of southern Sahelian landscapes, is a promising indicator for their establishment in the RFOROA.

Finally, the methods presented here are readily applicable to other terrestrial species capable of carrying GPS tracking devices, especially mobile animal species occupying highly seasonal environments. This approach may be particularly relevant for ongoing reintroductions of Sahelo-Saharan antelope, including oryx and addax in Tunisia and Morocco and addax in the RFOROA. Using population-level movements to delineate seasons may serve as a similar assessment of their habituation to local environmental conditions, and inform site-specific reintroduction and management practices.

## Supplementary Information

Additional file 1: Table S1. Summary of candidate model structures and model evaluation criteria. Models are ranked by the leave-one-out cross-validation information criterion (“looic”, calculated by the loo function in the loo package; Vehtari, Gelman, and Gabry 2017) where lower values indicate a better fit. The “p_loo” criteria assesses model complexity, and indicates how difficult it is to predict non-observed values (also calculated using loo). Additional criteria used to evaluate candidate models included: Bayesian R2 (“bayes_R2,” calculated by the bayes_R2 function in the brms package), LOO-adjusted R2 (“loo_R2”, calculated by loo_R2 in brms), and the Bayesian estimate of the expected log pointwise predictive density (“elpd_loo”, calculated by the loo function in the loo package) where larger ELPD values indicate a better fit. Figure S1. Diagnostic plots for the final model. (A) The empirical cumulative distribution function (ecdf) of the observed data (y, *dark blue line*) compared to that of the model-simulated data (yrep, *light blue line*; created using the *pp_check* function (type = “ecdf_overlay”) from the *bayesplot* package). (A) Posterior predictive check plot of the mean of the observed data (T(y), *dark blue line*) compared to means of model-simulated datasets (T(yrep), *light blue shading*). (C) Population-level mean movement rates and associated error estimates predicted by the final model (created using the *predict* function from the *brms* package). (D) PSIS diagnostic plot showing that all pareto k values for the final model < 0.5, indicating good model performance. Created using the *psis* object produced by the *loo* function in the *loo* package. ..Figure S2. Year weakly affects daytime movement rates by reintroduced oryx. Posterior density plot of the random effect of year included in the final model indicate that individual years generally have weak, non-significant effects on daytime movement rates by reintroduced oryx. The area under each curve shows the full posterior distribution for each random effect, *vertical blue lines* show the median estimate, and *light blue shading* shows 95% credible intervals. Created using the *mcmc_areas* function in the *bayesplot* package (Gabry et al. 2019). Figure S3. Reintroduced oryx exhibit high inter-individual variation in predicted daytime movement rates. Shaded areas show the posterior distribution of the random effect for each individual oryx, as estimated by the final model. *Vertical blue lines* indicate the posterior median, and *light blue shading* shows 95% credible intervals. Created using the *mcmc_areas* function in the *bayesplot* package (Gabry et al. 2019).

## Data Availability

The movement data analyzed in this study are not publicly available due to the conservation status and ongoing threats to the study population, but access may be requested from the Environment Agency—Abu Dhabi.
