# Supplementary material for: What is a season to an oryx? Movement rates identify three seasons for scimitar-horned oryx reintroduced into their native range
Source: Mov Ecol. 2025 Jul 29;13:56. doi: 10.1186/s40462-025-00536-7 (PMC12309194; doi:10.1186/s40462-025-00536-7)
Supplement: Supplementary file 1 — Additional file 1: Table S1. Summary of candidate model structures and model evaluation criteria. Models are ranked by the leave-one-out cross-validation information criterion (“looic”, calculated by the loo function in the loo package; Vehtari, Gelman, and Gabry 2017) where lower values indicate a better fit. The “p_loo” criteria assesses model complexity, and indicates how difficult it is to predict non-observed values (also calculated using loo). Additional criteria used to evaluate candidate models included: Bayesian R2 (“bayes_R2,” calculated by the bayes_R2 function in the brms package), LOO-adjusted R2 (“loo_R2”, calculated by loo_R2 in brms), and the Bayesian estimate of the expected log pointwise predictive density (“elpd_loo”, calculated by the loo function in the loo package) where larger ELPD values indicate a better fit. Figure S1. Diagnostic plots for the final model. (A) The empirical cumulative distribution function (ecdf) of the observed data (y, dark blue line) compared to that of the model-simulated data (yrep, light blue line; created using the pp_check function (type = “ecdf_overlay”) from the bayesplot package). (A) Posterior predictive check plot of the mean of the observed data (T(y), dark blue line) compared to means of model-simulated datasets (T(yrep), light blue shading). (C) Population-level mean movement rates and associated error estimates predicted by the final model (created using the predict function from the brms package). (D) PSIS diagnostic plot showing that all pareto k values for the final model < 0.5, indicating good model performance. Created using the psis object produced by the loo function in the loo package. ..Figure S2. Year weakly affects daytime movement rates by reintroduced oryx. Posterior density plot of the random effect of year included in the final model indicate that individual years generally have weak, non-significant effects on daytime movement rates by reintroduced oryx. The area under each curve shows the f [file 40462_2025_536_MOESM1_ESM.pdf]

Supplementary material

model name	bayes_R2	loo_R2	elpd_loo	p_loo	looic	model formula
m18	0.5640	0.5622	-68376.2	211.3	136752.5	log(movement rate) ~ s(day_of_year, reproductive_state, bs = "fs", xt = "cc") + s(age, by = reproductive_state) + reproductive_state + release_group + (1 | year) + (1 | animal_ID) + arma(time = date, gr = animal_ID, p = 5, q = 0)
m17	0.5638	0.5623	-68376.6	210.5	136753.2	log(movement rate) ~ s(day_of_year, reproductive_state, bs = "fs", xt = "cc") + s(age, by = reproductive_state) + release_group + (1 | year) + (1 | animal_ID) + arma(time = date, gr = animal_ID, p = 5, q = 0)
m19	0.5641	0.5622	-68376.8	212.6	136753.6	log(movement rate) ~ s(day_of_year, by = reproductive_state, bs = "cc") + reproductive_state + s(age, by = reproductive_state) + release_group + (1 | year) + (1 | animal_ID) + arma(time = date, gr = animal_ID, p = 5, q = 0)
m15	0.5639	0.5622	-68378.3	207.4	136756.7	log(movement rate) ~ s(day_of_year, reproductive_state, bs = "fs", xt = "cc") + s(age, by = reproductive_state) + reproductive_state + release_group + (1 | animal_ID) + arma(time = date, gr = animal_ID, p = 5, q = 0)
m14	0.5638	0.5622	-68378.9	206.7	136757.8	log(movement rate) ~ s(day_of_year, reproductive_state, bs = "fs", xt = "cc") + s(age, by = reproductive_state) + release_group + (1 | animal_ID) + arma(time = date, gr = animal_ID, p = 5, q = 0)
m16	0.5640	0.5621	-68379.0	208.8	136758.0	log(movement rate) ~ s(day_of_year, by = reproductive_state, bs = "cc") + reproductive_state + s(age, by = reproductive_state) + release_group + (1 | animal_ID) + arma(time = date, gr = animal_ID, p = 5, q = 0)
m12	0.5638	0.5621	-68379.2	210.1	136758.5	log(movement rate) ~ s(day_of_year, reproductive_state, bs = "fs", xt = "cc") + s(age, by = reproductive_state) + reproductive_state + (1 | animal_ID) + arma(time = date, gr = animal_ID, p = 5, q = 0)
m11	0.5637	0.5621	-68379.3	209.5	136758.6	log(movement rate) ~ s(day_of_year, reproductive_state, bs = "fs", xt = "cc") + s(age, by = reproductive_state) + (1 | animal_ID) + arma(time = date, gr = animal_ID, p = 5, q = 0)
m13	0.5637	0.5622	-68379.8	211.7	136759.6	log(movement rate) ~ s(day_of_year, by = reproductive_state, bs = "cc") + reproductive_state + s(age, by = reproductive_state) + (1 | animal_ID) + arma(time = date, gr = animal_ID, p = 5, q = 0)
m9	0.5631	0.5620	-68394.3	183.8	136788.6	log(movement rate) ~ s(day_of_year, reproductive_state, bs = "fs", xt = "cc") + s(age) + (1 | animal_ID) + arma(time = date, gr = animal_ID, p = 5, q = 0)
m10	0.5633	0.5620	-68394.6	185.2	136789.2	log(movement rate) ~ s(day_of_year, by = reproductive_state, bs = "cc") + reproductive_state + s(age) + (1 | animal_ID) + arma(time = date, gr = animal_ID, p = 5, q = 0)
m7	0.5619	0.5608	-68471.2	175.8	136942.3	log(movement rate) ~ s(day_of_year, reproductive_state, bs = "fs", xt = "cc") + release_group + (1 | animal_ID) + arma(time = date, gr = animal_ID, p = 5, q = 0)
m8	0.5621	0.5608	-68471.8	177.6	136943.5	log(movement rate) ~ s(day_of_year, by = reproductive_state, bs = "cc") + reproductive_state + release_group + (1 | animal_ID) + arma(time = date, gr = animal_ID, p = 5, q = 0)
m5	0.5617	0.5608	-68472.2	177.1	136944.4	log(movement rate) ~ s(day_of_year, reproductive_state, bs = "fs", xt = "cc") + (1 | animal_ID) + arma(time = date, gr = animal_ID, p = 5, q = 0)
m6	0.5619	0.5608	-68472.8	179.0	136945.7	log(movement rate) ~ s(day_of_year, by = reproductive_state, bs = "cc") + reproductive_state + (1 | animal_ID) + arma(time = date, gr = animal_ID, p = 5, q = 0)
m4	0.5607	0.5601	-68522.6	114.7	137045.2	log(movement rate) ~ s(day_of_year, bs = "cc") + (1 | animal_ID) + arma(time = date, gr = animal_ID, p = 5, q = 0)
m20	0.5552	0.5543	-68896.4	57.0	137792.7	log(movement rate) ~ reproductive_state + s(day_of_year, bs = "cc") + s(day_of_year, by = reproductive_state, bs = "cc") + arma(time = date, gr = animal_ID, p = 5, q = 0)
m3	0.5538	0.5534	-68950.0	23.6	137900.0	log(movement rate) ~ s(day_of_year, bs = "cc") + arma(time = date, gr = animal_ID, p = 5, q = 0)
m2	0.3646	0.3633	-79109.1	111.5	158218.2	log(movement rate) ~ s(day_of_year, bs = "cc") + (1 | animal_ID)
m1	0.1931	0.1929	-85898.8	12.0	171797.6	log(movement rate) ~ s(day_of_year, bs = "cc")

Table S1. Summary of candidate model structures and model evaluation criteria. Models are ranked by the leave-one-out cross-validation information criterion ("looic", calculated by the loo function in the loo package; (Vehtari, Gelman, and Gabry 2017) where lower values indicate a better fit. The "p_loo" criteria assesses model complexity, and indicates how difficult it is to predict non-observed values (also calculated using loo). Additional criteria used to evaluate

candidate models included: Bayesian R2 (“bayes_R2,” calculated by the bayes_R2 function in the brms package), LOO-adjusted R2 (“loo_R2”, calculated by loo_R2 in brms), and the Bayesian estimate of the expected log pointwise predictive density (“elpd_loo”, calculated by the loo function in the loo package) where larger ELPD values indicate a better fit.

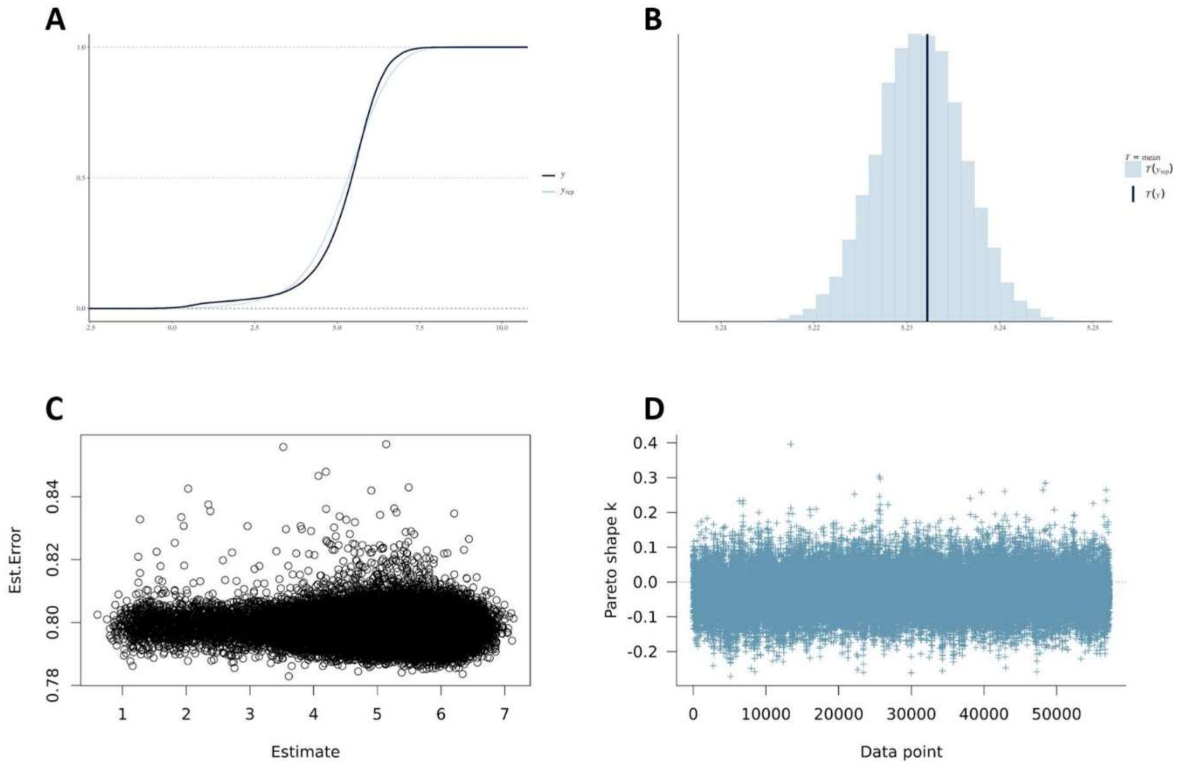

Figure S1. Diagnostic plots for the final model. (A) The empirical cumulative distribution function (ecdf) of the observed data (y, *dark blue line*) compared to that of the model-simulated data (yrep, *light blue line*; created using the *pp_check* function (type= "ecdf_overlay") from the *bayesplot* package). (A) Posterior predictive check plot of the mean of the observed data (T(y), *dark blue line*) compared to means of model-simulated datasets (T(yrep), *light blue shading*). (C) Population-level mean movement rates and associated error estimates predicted by the final model (created using the *predict* function from the *brms* package). (D) PSIS diagnostic plot showing that all pareto k values for the final model < 0.5, indicating good model performance. Created using the *psis* object produced by the *loo* function in the *loo* package.

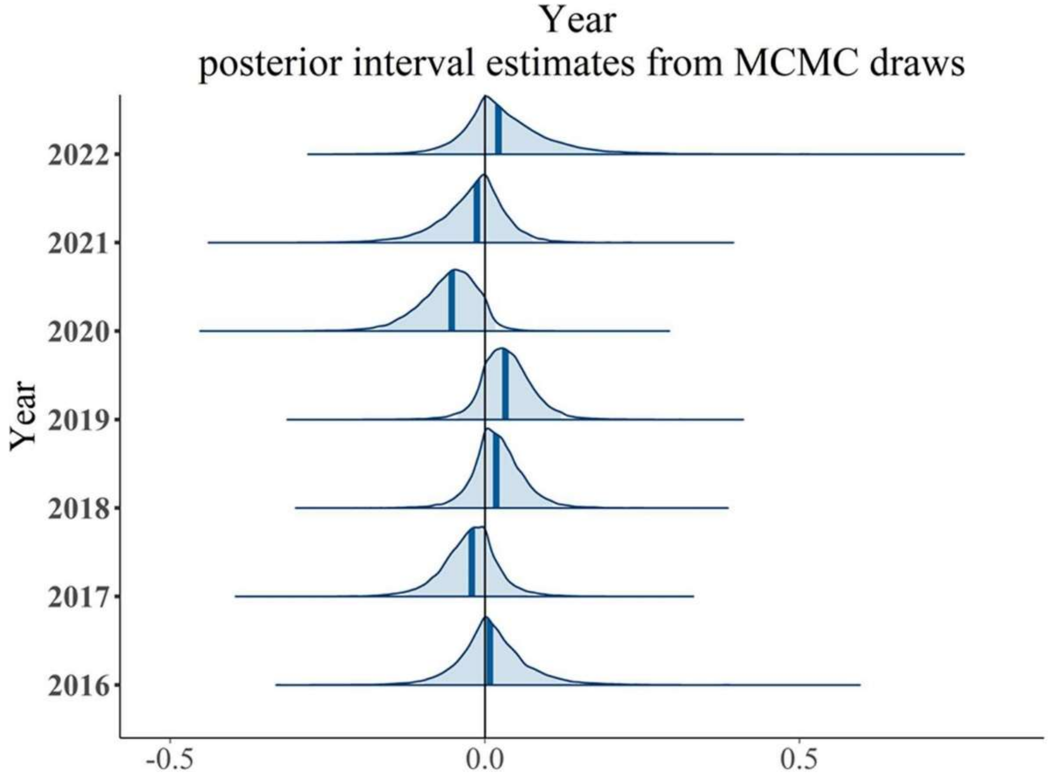

Figure S2. Year weakly affects daytime movement rates by reintroduced oryx. Posterior density plot of the random effect of year included in the final model indicate that individual years generally have weak, non-significant effects on daytime movement rates by reintroduced oryx. The area under each curve shows the full posterior distribution for each random effect, *vertical blue lines* show the median estimate, and *light blue shading* shows 95% credible intervals. Created using the *mcmc_areas* function in the *bayesplot* package (Gabry et al. 2019).

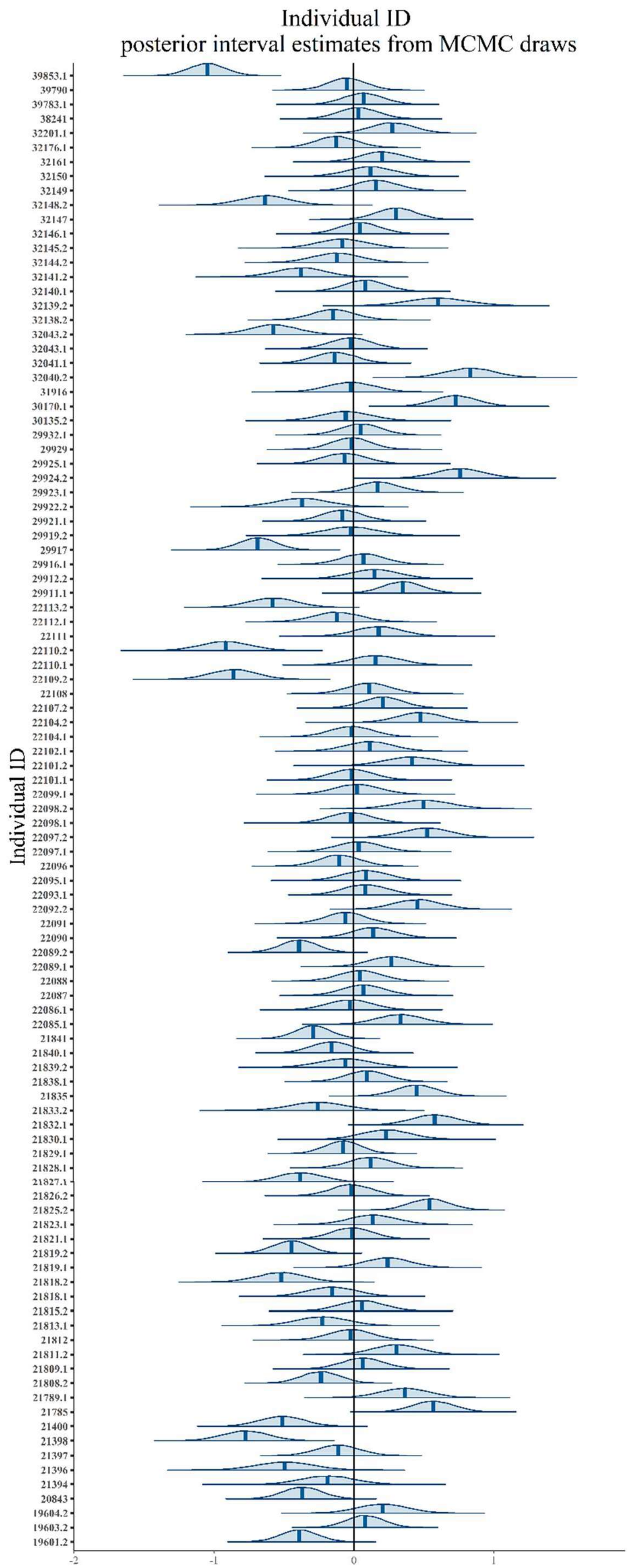

Figure S3. Reintroduced oryx exhibit high inter-individual variation in predicted daytime movement rates. Shaded areas show the posterior distribution of the random effect for each individual oryx, as estimated by the final model. *Vertical blue lines* indicate the posterior median, and *light blue shading* shows 95% credible intervals. Created using the *mcmc_areas* function in the *bayesplot* package (Gabry et al. 2019).
